# The other-race and other-species effects in face perception – a subordinate-level analysis

**DOI:** 10.3389/fpsyg.2014.01068

**Published:** 2014-09-19

**Authors:** Christoph D. Dahl, Malte J. Rasch, Chien-Chung Chen

**Affiliations:** ^1^Department of Psychology, National Taiwan UniversityTaipei, China; ^2^State Key Laboratory of Cognitive Neuroscience and Learning & IDG/McGovern Institute for Brain Research, Beijing Normal UniversityBeijing, China; ^3^Center for Collaboration and Innovation in Brain and Learning Sciences, Beijing Normal UniversityBeijing, China

**Keywords:** face perception, other-race effect, other-species effect, own-race advantage, own-species advantage, similarity, configural processing, heterospecific faces

## Abstract

The ability of face discrimination is modulated by the frequency of exposure to a category of faces. In other words, lower discrimination performance was measured for infrequently encountered faces as opposed to frequently encountered ones. This phenomenon has been described in the literature: the own-race advantage, a benefit in processing own-race as opposed to the other-race faces, and the own-species advantage, a benefit in processing the conspecific type of faces as opposed to the heterospecific type. So far, the exact parameters that drive either of these two effects are not fully understood. In the following we present a full assessment of data in human participants describing the discrimination performances across two races (Asian and Caucasian) as well as a range of non-human primate faces (chimpanzee, Rhesus macaque and marmoset). We measured reaction times of Asian participants performing a delayed matching-to-sample task, and correlated the results with similarity estimates of facial configuration and face parts. We found faster discrimination of own-race above other-race/species faces. Further, we found a strong reliance on configural information in upright own-species/-race faces and on individual face parts in all inverted face classes, supporting the assumption of specialized processing for the face class of most frequent exposure.

## INTRODUCTION

Humans as well as other primates are able to extract information from faces to infer invariant properties such as race (Michel et al., 2007; Tanaka and Pierce, 2009), species (Pascalis et al., 2002; Dufour et al., 2006), gender (Bulthoff, 2009), age (Kwon and da Vitoria Lobo, 1999), nationality (Li et al., 2004), rank (Dahl and Adachi, 2013), and to some extent personality traits (Ekman et al., 1980). The most important ability is to recognize and identify faces (Marr and Nishihara, 1978; Bruce and Young, 1986). Identity is an invariant face property that, however, is not fully independent from other invariant properties, such as race and species. Humans (Meissner et al., 2005; Hugenberg et al., 2010) and other primates, such as chimpanzees (Dahl et al., 2013a) and monkeys (Dahl et al., 2007, 2009, 2010, 2011) are experts in individuating the faces of their own species and race, however, their proficiency deteriorates for faces rarely exposed to. This phenomenon has been demonstrated by superior discrimination ability for the own-race as opposed to other-race faces (OREs), known as the other-race effect (ORE), the own-race advantage or the other-race bias (Lindsay et al., 1991; Meissner and Brigham, 2001). Similarly, there is an effect that it is easier to discriminate faces of the own species than those of other species. Such effect has been referred to as own-species advantage, the other- or own-species effect (OSE; Scott and Fava, 2013). The ORE has been addressed to a great extent (Meissner and Brigham, 2001) and elicited a robust effect in observers of different races (Bothwell et al., 1989). The OSE has been studied extensively in humans, using various behavioral (Dufour et al., 2004; Dufour and Petit, 2010) and neural methodological approaches (de Haan et al., 2002; Scott et al., 2005). Humans as well as monkeys show an advantage for own-species faces: they were more skilled in discriminating (Pascalis and Bachevalier, 1998) and recognizing (Dufour et al., 2004) the own-species as opposed to the other-species face (OSE) class. Human adults show better detection of configural differences in facial features of human but not monkey faces. Only inverted human but not monkey faces disrupt this sensitivity (Mondloch et al., 2006).

Still, it is not clear whether the two effects can be described by the same underlying computational mechanism of the face recognition system when it is exposed to a face class other than its own. In detail, the ORE occurs at the level of two face categories that are slightly different from each other in terms of facial components and configurations (shared human morphology), while the OSE occurs at the level of two face categories that fundamentally vary in the morphological structure. This raises an interesting question: do we recruit the same processing mechanism for other-race as for own-race faces and for OSEs as for own-species faces? Does the morphological (dis)similarity influence the configural processing of these face types? We examine the ORE and the OSE using the same paradigm embedded in the same experimental procedure. In a delayed matching-to-sample task, human participants discriminated pairs of faces of Asian and Caucasian races as well as chimpanzee, rhesus macaque and marmoset species. We hypothesize that any morphological deviation from the default class of faces (here Asian faces) causes a disruption of discrimination performance relative to the default class. We further estimated the contribution of configural as opposed to part-based contributions by correlating discrimination performances with the similarity values drawn from pairwise comparisons of the stimuli’s configural arrangement (configuration) and facial parts (part-based). Hence, we further hypothesize that the processing of own-class faces involves a relatively higher contribution of configural information as opposed to other face classes, while with increasing morphological distance from the conspecific face class, the contribution of part-based information, as in object-like processing, should increase. Further, assuming that face inversion impairs configural processing (Freire et al., 2000), we predict that inverted face comparisons of all classes show comparable performances that go along with an equal contribution of part-based information across all face classes.

## MATERIALS AND METHODS

### PARTICIPANTS

Twenty-four human participants (10 females; 22.5 years +/-1.5 SD) took part in this study. Twenty-two participants participated in the task using upright and 22 using inverted stimuli. In other words, two participants did not participate in both experiments. The participants were selected from a pool of students at the National Taiwan University and consist of Asian members exclusively. The participants have never encountered the stimuli used and they have never been tested on a comparable face discrimination task. The participants were naïve to the purpose of the experiment. Written consent was obtained from each participant prior to the experiment.

### APPARATUS AND PROCEDURE

We used a delayed-matching-to-sample paradigm (DMS), presenting one face stimulus centrally on the display for 300 ms (*cue*), followed by an inter-stimulus interval (ISI) of 250 ms, followed by a mask for 300 ms, followed by a second ISI of 500 ms and two face stimuli, with one showing the same picture as in the cue presentation (*match*) and one showing a different individual’s face picture (*distractor*). The *match* and *distractor* were shown for 3500 ms, however, responses could be given after disappearance of the stimuli on the display. The task was to indicate which of the two stimuli (*match* and *distractor*) is identical to the *cue* stimulus. In the inversion experiment, all faces, including the cue, were rotated in image-plane for 180°. The vertical spacing of the match and distractor stimuli was about 40 mm (2.92° of gaze angle). Stimuli were presented at a 19-inch CRT display (1024 × 768 pixels) controlled by custom-written software under MATLAB (Mathworks Inc., Natick, MA, USA) and Psychtoolbox (Brainard, 1997; Pelli, 1997). Testing of upright and inverted stimuli was done block-wise and separated by maximally 3 days. Participants received financial compensation. Participants sat in an experimental room (2.5 m wide, 2.5 m deep), facing the computer monitor at a viewing distance of 100 cm. The viewing distance was controlled using a chin rest.

### STIMULI

We used black-and-white pictures of faces of Asian and Caucasian humans as well as chimpanzees (*Pan troglodytes*), Rhesus macaques (*Macaca mulatta*) and Marmoset monkeys (*Callithrix jacchus*). All faces were of unfamiliar individuals. All stimuli were normalized for luminance and contrast and presented on the screen in an image canvas of 5.75 by 5.75° of gaze angle. Masks were created by extracting 50 rectangular patches from the original face stimuli. The size of these patches was 13.33% of the original image dimensions. Patches were randomly placed onto a white blank surface (a canvas of the same size as the face stimuli). This procedure resulted in certain overlap of individual patches, while fully covering the blank surface. For each face category, mask stimuli were created and solely used for trials of the same type of faces. In total, we used five sets of 12 face stimuli each, resulting in 660 comparisons. In each trial, the mask was randomly chosen from a set of 50 masks. Each participant did five runs of 132 trials each consisting of all five categories intermixed and fully counter-balanced across categories and face identities.

### DATA ANALYSIS

The analyses were performed using Matlab (Mathworks Inc., Natick, MA, USA). The dependent variable was reaction times and error rates. Trials with reaction times above 4000 ms were excluded. Only correct trials went into the analyses of reaction times, which is 547 (+/-8.26 SD) upright and 553 (+/-10.7 SD) inverted trials. The statistics follow a within-subject design. Analyses of variances were performed using a fixed effect ANOVA with stimulus category and stimulus presentation (upright, inverted) as fixed factors, an interactive analysis of covariance (ANCOVA) and two-sample *t*-tests. The similarity scores of the facial configuration within face classes were determined by calculating the mean of the Euclidean distances between corresponding configural marker points of each pair of faces. These markers were placed manually by the authors. Euclidean distance were further visualized by classical multidimensional scaling (cMDS), which aims at placing each face in *N*-dimensional space such that the between-face distances are preserved as well as possible. The maximum error is 2.68 in a two-dimensional and 2.66 in a full reconstruction. To determine the similarity between individual facial parts (eyes, nose, mouth), we used topological methods (Gunduz and Krim, 2003) that viewed each two-dimensional images as a surface with the pixel intensities represented as values on the *z*-axis. Using the second derivatives the principle curvatures of the surface were extracted. The facial features, such as eyes, nose, and mouth, were composed of valleys and crests. Areas of interests were determined by cutting the image in the horizontal dimension. The cut-off lines varied across face classes given the differential face morphology. The extracted curvature profiles were average in the horizontal dimension for each image, resulting in a feature vector. The feature vectors of individual parts were normalized across all classes to an equivalent length, i.e., number of samples. Similarity scores of facial parts were correlated with the reaction times for upright and inverted face trials separately and similarity scores of the facial configuration were correlated with the performance difference of upright and inverted face trials by subtracting the upright face trials from the inverted ones.

## RESULTS

**FIGURE 1:**
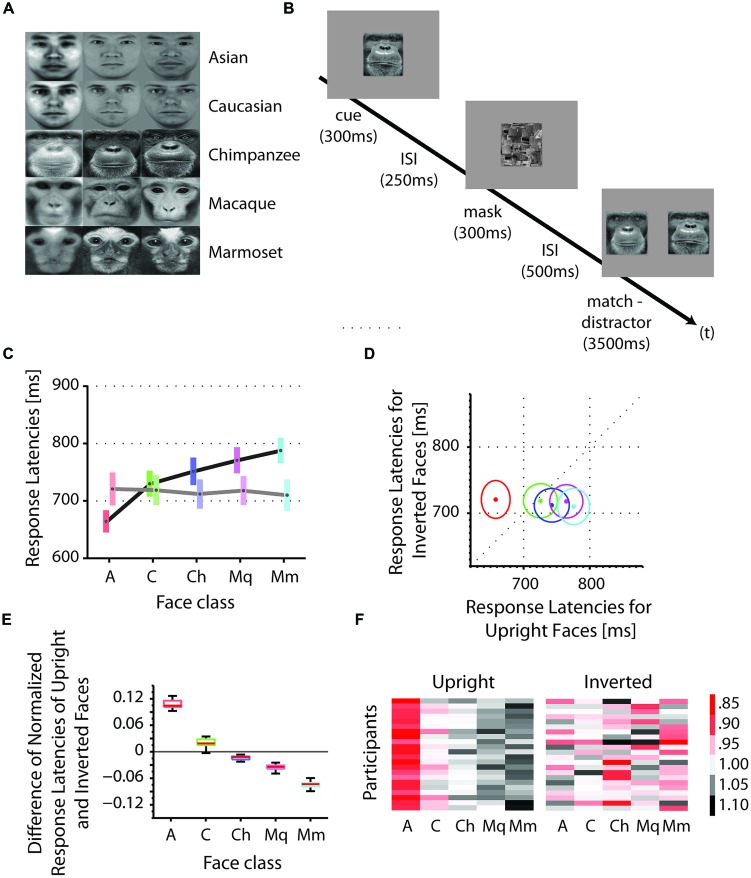
**Stimuli, procedure and reaction times, and configural analysis. (A)** Stimuli: identities (horizontal) and classes (vertical). **(B)** Procedure. In each trial, a face picture of an individual (cue) was centrally presented on the display for 300 ms, followed by an inter stimulus interval (ISI) of 250 ms, followed by a mask for 300 ms, followed by a second ISI of 500 ms and a presentation of two horizontally aligned face pictures of the same individual (match) and a different individual (distractor). Participants indicated their choice by pressing one of two buttons on the joystick, corresponding to the left and right stimulus on the screen. The correct answer (match) is the identical face picture as shown in the cue stimulus. **(C)** Absolute reaction times were averaged across participants for each face class (A, Asian; C, Caucasian; Ch, chimpanzee; Mq, macaque; Mm, marmoset). The color bars indicate the range within the standard errors; the gray dots indicate the means. Reaction times for upright faces are shown in darker colors; those for inverted faces in lighter colors. **(D)** Absolute reaction times of upright versus inverted faces. Mean reaction times for upright and inverted faces are shown as dots. Colors correspond to the colors in **(C)**. Circles indicate the standard errors. **(E)** Differences of normalized reaction times of upright and inverted faces. Reaction times were normalized for each face class by the mean of the corresponding class. Scores for upright faces were subtracted from those of inverted faces for each face class. Positive values indicate an advantage for upright above inverted faces in terms of discrimination speed, while negative values indicate an advantage for inverted above upright faces. **(F)** Normalized reaction times for each participant. The color code represents the relative reaction times, with red being fast, black being slow and white intermediate. Values above zero indicate slower responses than the mean response (= 0); values below zero indicate faster responses than the mean response.

We tested the reaction times of Asian participants on the discrimination of Asian and Caucasian human faces, as well as chimpanzee (*P. troglodytes*), macaques (*M. mulatta*) and marmoset monkey (*C. jacchus*) faces in upright and inverted orientations (**Figure 1A**). A cue stimulus (e.g., face 1) was centrally presented followed by a match-distractor stimulus pair (e.g., face 1 and face 2) of the same species (**Figure 1B**). We collected an average of 660 trials for upright as well as inverted presentations in 22 participants. Using a fixed effect ANOVA with stimulus class and face orientation (upright, inverted) as fixed factors and reaction times as dependent variable, we found a significant interaction of the factors stimulus class and the face orientation [*F*(4,210) = 2.52, *p* < 0.05, mean square error = 29388; **Figures 1C–E**]. In more detail, we found a systematic facilitation of discrimination for upright own-race faces (Asians) as opposed to upright OREs [Caucasians; *t*(42) = -2.22, *p* < 0.05; mean *Asians* = 660.7 ms, mean *Caucasians* = 726.2 ms; one-tailed]. Similarly, we found an advantage for the same upright Asian faces (own-species) as opposed to upright other-species’ faces, such as chimpanzee, macaques and marmosets [*t*(86) = -4.16, *p* < 0.001; mean *Asians* = 660.7 ms, mean *chimpanzee* = 748.9 ms, mean *macaque* = 767.5 ms, mean *marmoset* = 784.5 ms; one-tailed, corrected for multiple comparisons]. These two effects, the own-race advantage and the own-species advantage, do not appear when the face stimuli were presented inverted (180° image plane rotation): Asian faces (own-race, own-species) are processed at the same speed as Caucasian faces [other-race; *t*(42) = 0.05, *p* = 0.96; mean *Asians* = 720.7 ms, mean *Caucasians* = 718.7 ms] as well as OSEs [*t*(86) = 0.25, *p* = 0.81; mean *Asians* = 720.7 ms, mean *chimpanzee* = 712 ms, mean *macaque* = 717.9 ms, mean *marmoset* = 710 ms]. At the same time, inversion caused a significant increase of reaction times for Asian faces [own-race, own-species; *t*(42) = -1.74, *p* < 0.05; mean upright *Asians* = 660.7 ms, mean inverted *Asians* = 720.7 ms; one-tailed], but no change in reaction times for Caucasian [other-race; *t*(42) = 0.22, *p* = 0.83; mean upright *Caucasians* = 726.2 ms, mean inverted *Caucasians* = 718.7 ms], chimpanzee [other species; *t*(42) = 1.05, *p* = 0.30; mean upright *chimpanzee* = 748.9 ms, mean inverted *chimpanzee* = 712 ms] and macaque faces [other species; *t*(42) = 1.46, *p* = 0.15; mean upright *macaque* = 767.5 ms, mean inverted *macaque* = 717.8 ms]. Inverted presentations of marmoset faces (other species), however, caused a facilitation in comparison to the upright presentations of the same faces [*t*(42) = 2.12, *p* < 0.05; mean upright *marmoset* = 784.5 ms, mean inverted marmoset = 710 ms].

We further examined relative changes of reaction times by normalizing the data samples for each face class by dividing them with the grand mean of all face classes. We found that the scores decreased with evolutionary distance from Asian faces: (1) scores for Asian faces were greater than those for Caucasian faces, reflecting the ORE [*t*(42) = 30.70, *p* < 0.001; mean scores *Asian* = 0.11; mean scores *Caucasian* = 0.02]; (2) scores for Caucasian faces were greater than those for chimpanzee faces [*t*(42) = 9.64, *p* < 0.001; mean scores *Caucasian* = 0.02; mean scores *chimpanzee* = -0.02]; (3) scores for chimpanzee faces were greater than those for macaque faces [*t*(42) = 4.62, *p* < 0.001; mean scores *chimpanzee* = -0.02; mean scores *macaque* = -0.04]; and (4) scores for macaque faces were greater than those for marmoset faces [*t*(42) = 14.34, *p* < 0.001; mean scores *macaque* = -0.04; mean scores *marmoset* = -0.07]. This trend is consistent across all participants (**Figure 1F**).

Using an interactive ANCOVA with stimulus class and face orientation (upright, inverted) as fixed factors, error rates as covariate, and reaction times as dependent variable, we found no significant effects of error rates on stimulus class and face orientation (all *p*-values > 0.26). Hence, we rule out a speed-accuracy trade-off. For further analyses we focused on reaction times.

**FIGURE 2:**
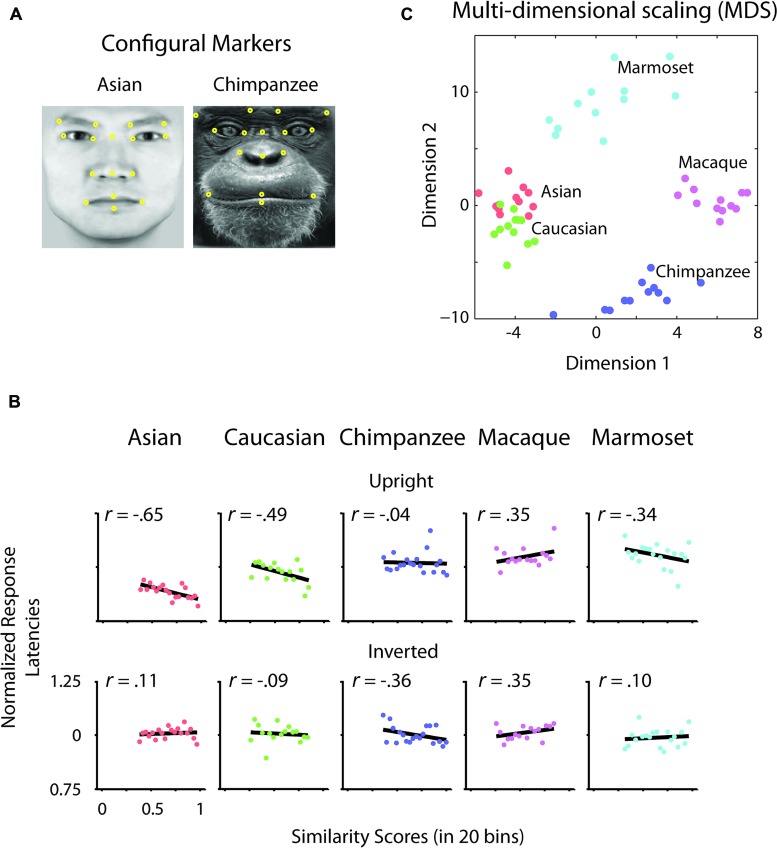
**Configural analysis. (A)** Configural markers. Illustrative arrangement of facial markers on Asian and chimpanzee faces (yellow dots). Corresponding points as shown were selected for each individual face. **(B)** Differences of normalized reaction times of upright and inverted faces as a function of configural similarity. Differences of normalized reaction times of upright and inverted faces were correlated with the binned values of similarity scores drawn from the configural arrangement of pairs of faces. Black lines indicate the correlation. **(C)** Multidimensional scaling (MDS) space of configural similarities among faces and face classes.

**FIGURE 3:**
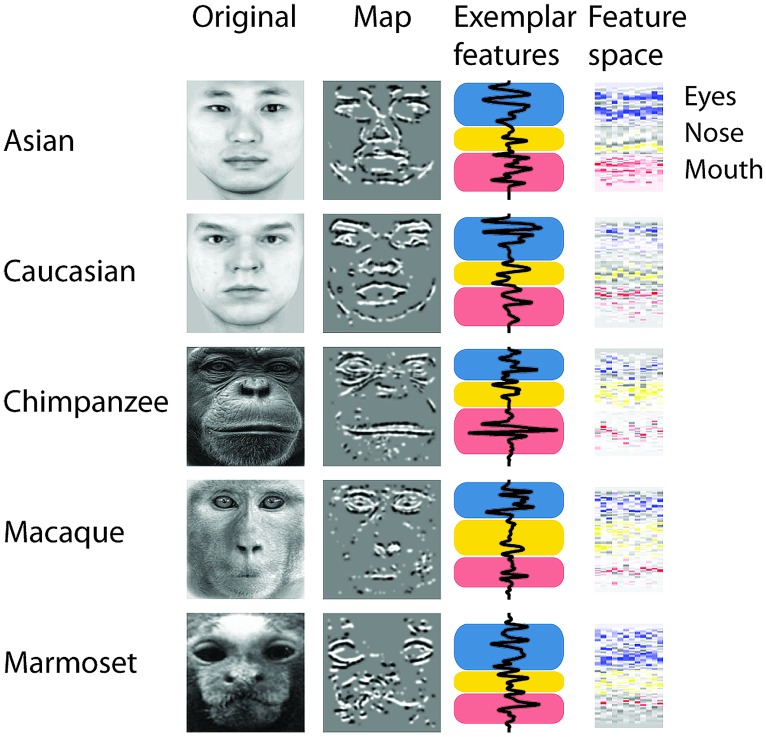
**Face part extraction, part-based analysis.** The process of extraction face parts from an original image (non-normalized images are shown here for illustration). Black and white colors in the “Map” plots illustrate the ravines of the face. Colors in the “Exemplar features” and “Feature space” plots illustrate the face region (blue = eyes; yellow = nose; red = mouth). The “Feature space” panels show the feature profiles (*y*-axis) for each stimulus (*x*-axis).

**FIGURE 4:**
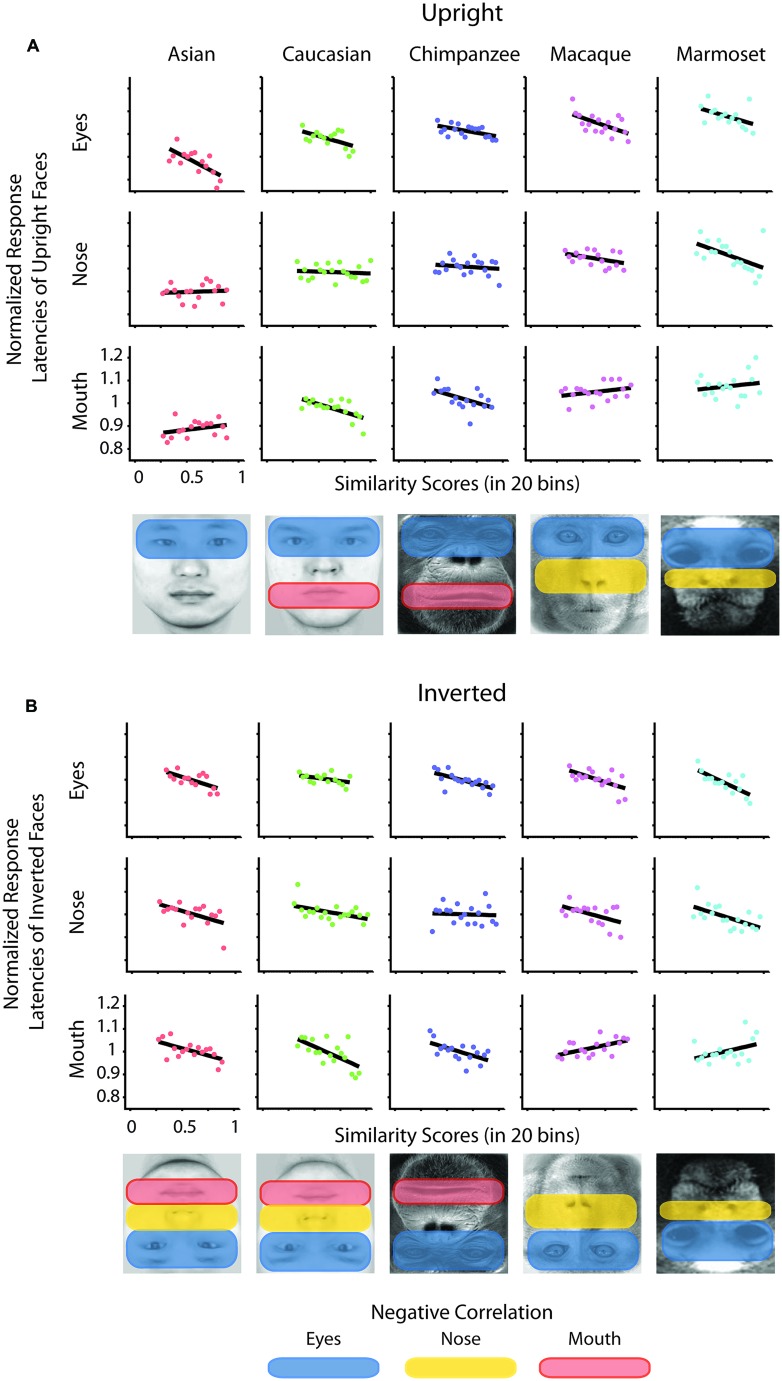
**Normalized reaction times of upright and inverted faces as a function of similarity scores drawn from face parts.** Normalized reaction times of upright **(A)** and inverted faces **(B)** were separately correlated with the binned values of similarity scores drawn from the face parts of pairs of faces. Black lines indicate the correlation. Below the graphs the significantly negatively correlated areas are highlighted.

To determine whether there is a relation between the similarity of a stimulus pair and the discrimination performances we calculated similarity values for each face stimulus based on (1) the facial configuration and (2) individual parts (eyes, nose, and mouth). (1) We determined configural similarities among individual faces of the same face classes by calculating the Euclidean distances between corresponding marker-points set in the 2D face plane (**Figure 2A**). These values reflect the overall structure of the face, but do not include any image information. We determined similarity values between individual face configurations. These values were then correlated with the corresponding reaction times for each face class separately. For each participant reaction times were normalized by the grand mean and assigned to 20 bins of equal sizes ranging from the minimal to the maximal sample value. We analyzed upright and inverted faces separately. A negative correlation between reaction times and similarity of facial configuration would indicate that to discriminate two faces is more difficult the more similar two face configurations are. We found a negative correlation between the normalized reaction times of upright faces and the similarity scores in Asian (*r* = -0.65, *p* < 0.01) and Caucasian faces (*r* = -0.49, *p* < 0.05), but not in any OSE class (chimpanzee *r* = -0.04, *p* = 0.85; macaque *r* = 0.35, *p* = 0.18; marmoset *r* = -0.34, *p* = 0.16; **Figure 2B**). We did not find any negatively correlated relationship between the normalized reaction times of inverted faces and the similarity scores in any of the face classes (all *p* > 0.36; **Figure 2B**). (2) We further extracted face features using methods suggested in computer vision (Gunduz and Krim, 2003). This approach treats the face image as a surface with eyes, nose and mouth being singularities in the surface that build valleys and ridges of the luminance landscapes (**Figure 3**). Using so-called ravines facial features can be extracted from the surface. We extracted eyes, nose and mouth regions and compared the vector profiles of these features among the faces of each face class. We determined the Euclidean distances between corresponding points of the two vector profiles being compared and correlated these similarity scores with the normalized reaction times of upright and inverted face discrimination trials (**Figures 4A,B**). Importantly, in contrast to the configural similarity, the part similarity was determined by actual image content, i.e., vector profiles were extracted from the image surface. The assumption is that a negative correlation between reaction times and similarity values for a certain face part would occur, if participants strongly rely on that face part and, thus, are negatively affected if two faces have closely similar parts. We found negative correlations between the eyes and the similarity scores for all classes when the faces were presented upright (Asian: *r* = -0.69, *p* = 0.01; Caucasian: *r* = -0.55, *p* < 0.05; chimpanzee *r* = -0.54, *p* < 0.01; macaque *r* = -0.59, *p* < 0.01; marmoset *r* = -0.47, *p* = 0.05). The similarity scores of the nose regions were not correlated with reaction times of upright faces in all face classes, but for the macaque and marmoset monkeys (Asian: *r* = 0.08, *p* = 0.62; Caucasian: *r* = -0.11, *p* = 0.33; chimpanzee *r* = -0.16, *p* = 0.25; macaque *r* = -0.41, *p* < 0.05; marmoset *r* = -0.52, *p* = 0.01). The similarity scores of the mouth region were negatively correlated with the reaction times of upright face in Caucasian and chimpanzee faces (Asian: *r* = 0.29, *p* = 0.88; Caucasian: *r* = -0.64, *p* < 0.001; chimpanzee *r* = -0.52, *p* < 0.05; macaque *r* = 0.29, *p* = 0.89; marmoset *r* = 0.18, *p* = 0.76). In contrast, the similarity scores of all parts were negatively correlated with the reaction times of inverted faces with some exceptions: the chimpanzee’s nose and the macaque’s and marmoset’s mouths did not elicit any correlation (Eyes: Asian: *r* = -0.65, *p* < 0.001; Caucasian: *r* = -0.43, *p* < 0.05; chimpanzee *r* = -0.65, *p* < 0.01; macaque *r* = -0.60, *p* < 0.01; marmoset *r* = -0.74, *p* = 0.001; Nose: Asian: *r* = -0.54, *p* < 0.05; Caucasian: *r* = -0.48, *p* < 0.05; chimpanzee *r* = -0.06, *p* = 0.40; macaque *r* = -0.48, *p* < 0.05; marmoset *r* = -0.59, *p* < 0.01; Mouth: Asian: *r* = -0.61, *p* < 0.01; Caucasian: *r* = -0.65, *p* < 0.01; chimpanzee *r* = -0.57, *p* < 0.01; macaque *r* = 0.56, *p* = 0.99; marmoset *r* = 0.40, *p* = 0.95).

We found a relative increase of reaction times for the discrimination of OREs as well as OSEs. The OSE for upright faces becomes more pronounced with increasing distance from the human species on the evolutionary timeline. We further showed that while face inversion has a marginal or facilitative effect on faces of the other race, it has a drastic deteriorative effect on faces of the same race (see discussion). Same-race faces showed a strong negative correlation between reaction times and configural similarity, indicating configural sensitivity to a great extent. OREs showed a relatively weaker negative correlation between reaction times and configural similarity, indicating weaker configural sensitivity. All other faces showed negative correlations between reaction times and similarity scores of certain facial parts, suggesting processing mechanisms with focus on individual – likely the most distinctive – face parts.

## DISCUSSION

We addressed how configural processing and similarity at subordinate-level (within-class) affect discrimination performance for faces. Only a few accounts, to date, have taken similarity among stimulus samples into consideration when evaluating discrimination performances (Martin-Malivel and Okada, 2007; Dahl et al., 2013a). Here we evaluate the extent to which participants rely on configural similarity among the two samples of a stimulus pair. A related study showed that with increasing similarity among stimuli configural processing increased (Hsiao and Cheung, 2011). We determined the reliance on configural processing by looking at upright as opposed to inverted faces. The results indicate that participants relied on configural information more strongly with increasing configural similarity between the two faces presented. This was true only for the own face class, to a lesser degree for the ORE class and only in upright presentation condition. In other words, the more similar the configurations of two faces was the more the system relied on configural processing in order to successfully discriminate the two faces.

We also addressed the extent to which the participants relied on specific facial parts. We expect faces of other species and races as well as inverted own-race and own-species faces to be processed in a stronger part-based manner than upright own-species and own-race faces. The part-based analysis revealed a general relationship between similarity of parts and discrimination performance for all face classes. In more detail, the eyes played a crucial role for all types of classes; the mouth played a more important role for Caucasian and chimpanzee faces, not so much for monkey faces; while in contrast the nose played a more critical role in monkey faces as opposed to human and chimpanzee faces. As can be seen in **Figure 1A**, left column, the chimpanzee faces, in contrast to the monkey faces, contain a large variance in the mouth area. In other words, differential diagnostic features are used for the OSE classes. In the upright own-face class, only the similarity of eyes was negatively correlated with the performances, which might be caused by the strong eye dominance in face recognition, as described in humans (Barton et al., 2006) and monkeys (Keating and Keating, 1993; Gothard et al., 2004; Dahl et al., 2007). This, however, drastically changes when the own-class faces were presented inverted: similarity of all parts were correlated negatively with the performance scores, speaking for a rather distributed account on discriminating inverted own-race/species faces. Together, these findings consistently reflect the involvement of two processing strategies, configural and part-based processing, depending on the type of face and the presentation condition. Along the lines, eye tracking studies in humans (Barton et al., 2006) and monkeys (Dahl et al., 2009) showed that eye gaze distribution were more compact on the eye region of upright conspecific faces and more distributed across facial parts for upright non-conspecific and inverted faces. Here, we found stronger focus on the eyes only in the upright own-race/species faces and more distributed reliance on several facial parts for upright other-race/species faces as well as inverted faces. In combination with the stronger reliance on configural similarity for upright own-race/species faces, we can disentangle the two processing mechanisms.

How can we interpret our findings? A critical component in face perception is the level of expertise the observer has with the face class presented. Expertise of the observer was found to influence ORE. Configural information is used to a greater extent for own-race as opposed to OREs (Tanaka et al., 2004; Michel et al., 2006): Caucasians showed a larger whole-face advantage (in comparison to using individual facial parts) for own-race as opposed to OREs, while Asians, living in a society predominantly populated by Caucasians, showed an equal whole-face advantage for both types of faces. Similarly, expertise influences the OSE: humans and monkeys showed higher sensitivity toward so-called second-order relational properties (Adachi et al., 2009; Dahl et al., 2010, 2011), i.e., the spatial dimensions among facial parts (Tanaka and Farah, 1991), and diagnostic scanning patterns indicating configural processing of own-species faces and part-based processing of OSEs (Dahl et al., 2009). Accordingly, adult humans more easily detect slight spatial changes in human faces relative to monkey faces, while the performance is impaired for human faces when presented in inverted condition (Mondloch et al., 2006). Further, captive chimpanzees were tested on discriminating chimpanzee and human faces (Dahl et al., 2013a). Young chimpanzees (around 10 years of age) showed a clear advantage for chimpanzee faces above human faces; however, the advantage turned into a disadvantage with increasing exposure to human faces and limited exposure to chimpanzee faces: older chimpanzees (around 30 years of age) showed an advantage for human above chimpanzee faces. In other words the sensitivity toward one class of faces adapted toward another class of faces more strongly exposed to over decades. These same chimpanzees showed a more pronounced face inversion effect (Dahl et al., 2013b) and a more pronounce left-chimeric face bias (indicating dominant right-hemispheric processing; Dahl et al., 2013c) for faces of the expert category. Monkey experts turn out to be more accurate than non-experts at identifying faces of expertise and were more affected by inversion of those monkey faces (Dufour and Petit, 2010).

As shown in this study, the similarity of two faces influences how fast we can differentiate the two. In terms of the own-race face configuration, the more closer the configural arrangements of two faces are, the more the observer relied on a configural approach. While we report an own-race advantage in terms of reaction times, i.e., faster discrimination for own-race than OREs, we find that configural information plays a role in both own-race as well as OREs. However, it needs to be clearly stated that the effect size is greater for own-race than OREs, with the latter being significant at exactly 5%. In other words, a face class which is morphologically close to the own-face class can be treated to some extent configurally [see **Figure 2C** for morphological similarity in a multidimensional scaling (MDS) space]. However, with increasing morphological distance OSEs, configural information cannot be successfully used for face discrimination. In accordance with this interpretation, own-race faces have been reported to be processed more configurally than OREs (Michel et al., 2006). Further, according to the expertise (contact) hypothesis (Bukach et al., 2006), the extent to which OREs were accessed configurally, might well reflect the level of exposure of our participants to Caucasian faces. Critically, our results suggest that there is no on-off state between part-based and configural processing strategies, but rather a continuous change of processing strategies along the grades of similarity with own-class faces.

How can we explain the trend toward facilitation of primate faces when inverted as opposed to upright? First, it has to be clearly stated that there was no significant inversion effect for face classes of the closer evolutionary relatives to humans, chimpanzees and macaques. However, marmoset monkey faces showed a difference, and chimpanzee and macaque faces showed a trend toward a face inversion effect. A possible explanation is as follows: when the non-human primate faces were presented upright, the visual system attempts to process these faces like human (here Asian) faces due to a default approach to face-like stimuli. However, given the high degree of morphological difference between non-human primate and human faces, default-template processing fails for non-human primates to a great extent. This would explain an increasing deterioration with increasing evolutionary distance. Once the faces were presented inverted, they were all treated equally and processed according to a part-based manner, hence resulting in relatively equal reaction times for discriminating human and non-human primate faces. In a recent computational model (SCORE; Dahl et al., accepted), we found that, indeed, the representational structure of Asian faces was more different in its higher dimensional components to the representational structure of chimpanzee faces than Caucasian faces. This supports the idea that applying a human face template onto a non-human face reduces the discrimination power drastically.

Face perception is governed by two processing mechanisms, configurally, and part-based processing, and presumably a mixture of both. This study shows that own-class faces tend to follow configural processing rules, while other face classes are governed more strongly by part-based processing. The interplay of configural and part-based processing is influenced by a number of factors: (1) Expertise in discrimination faces drives the perceptual system toward a configural strategy. (2) Morphological similarity among face classes determines the extent to which the perceptual system is able to generalize from the expert face class to a non-expert face class. In our study, Asian-face experts can to some degree make use of configural information in Caucasian faces due to the close morphological distance of Asian and Caucasian faces. (3) Inversion disrupts access to configural information largely and breaks down the processing strategies of all face classes to an analytic part-based manner. Hence, these factors influence the general discrimination ability of various face classes and manifest in effects like the ORE and OSE.

## AUTHOR CONTRIBUTIONS

Christoph D. Dahl conceived, designed and performed the experiments; Christoph D. Dahl analyzed the data; Christoph D. Dahl, Malte J. Rasch and Chien-Chung Chen contributed reagents/materials/analysis tools; Christoph D. Dahl wrote the paper. All authors discussed the results and commented on the manuscript.

## Conflict of Interest Statement

The authors certify that they have no affiliations with or involvement in any organization or entity with any financial interest, or non-financial interest in the subject matter or materials discussed in this manuscript.

## APPROVING COMMITTEE

The study was approved by the Research Ethic Committee of National Taiwan University and followed the principles of Helsinki Declaration and local laws and regulations.
