# Erratum on: The other-race and other-species effects in face perception—a subordinate-level analysis

**DOI:** 10.3389/fpsyg.2015.00010

**Published:** 2015-01-21

**Authors:** Frontiers Production Office

**Affiliations:** Frontiers Production OfficeFrontiers, Switzerland

**Keywords:** face perception, other-race effect, other-species effect, own-race advantage, own-species advantage, similarity, configural processing, heterospecific faces

Reason for Erratum:

The affiliation for the authors Christoph D. Dahl and Chien-Chung Chen was listed as Department of Psychology, National Taiwan University, Taipei, China instead of Department of Psychology, National Taiwan University, Taipei, Taiwan, due to a typesetting error. This mistake does not change the scientific conclusions of the article in any way.

The publisher apologizes for this error.

